# DNA6mA-MINT: DNA-6mA Modification Identification Neural Tool

**DOI:** 10.3390/genes11080898

**Published:** 2020-08-05

**Authors:** Mobeen Ur Rehman, Kil To Chong

**Affiliations:** 1 Department of Electronics and Information Engineering, Jeonbuk National University, Jeonju 54896, Korea; cmobeenrahman@gmail.com or; 2 Department of Avionics Engineering, Air University, Islamabad 44000, Pakistan; 3 Advanced Electronics and Information Research Center, Jeonbuk National University, Jeonju 54896, Korea

**Keywords:** DNA N6-methyladenine, Chou’s 5-steps rule, Convolution Neural Network (CNN), Long Short-Term Memory (LSTM), computational biology

## Abstract

DNA N${}^{6}$-methyladenine (6mA) is part of numerous biological processes including DNA repair, DNA replication, and DNA transcription. The 6mA modification sites hold a great impact when their biological function is under consideration. Research in biochemical experiments for this purpose is carried out and they have demonstrated good results. However, they proved not to be a practical solution when accessed under cost and time parameters. This led researchers to develop computational models to fulfill the requirement of modification identification. In consensus, we have developed a computational model recommended by Chou’s 5-steps rule. The Neural Network (NN) model uses convolution layers to extract the high-level features from the encoded binary sequence. These extracted features were given an optimal interpretation by using a Long Short-Term Memory (LSTM) layer. The proposed architecture showed higher performance compared to state-of-the-art techniques. The proposed model is evaluated on *Mus musculus*, Rice, and “Combined-species” genomes with 5- and 10-fold cross-validation. Further, with access to a user-friendly web server, publicly available can be accessed freely.

## 1. Introduction

In genomes of distinct species, DNA N${}^{6}$-methyladenine (6mA) illustrates a crucial epigenetic transformation [1,2]. DNA 6mA is a non-canonical process that modifies the catalyzed adenine ring of DNA methyltransferases [3]. Alteration occurs at the sixth position of the adenine ring where a methyl group is additionally introduced. DNA 6mA holds a vital role in numerous biological processes, which includes DNA replication [4], DNA repair [5], DNA transcription [6], and others. Recent research established that uneven 6mA modification has a role in different diseases such as cancer [7], immune systems, and others. Therefore, this makes it necessary to identify a 6mA position in the genome sites. Mammalian 6mA largely originates from the genomic incorporation mediated by DNA polymerase, while the methylase-generated 6mA in mice remains elusive [8].

Silico prediction is considered to be a principal approach to encounter the aforementioned problem, while N${}^{6}$-methyladenine prediction is its alternative. Intensive labor with extravagant experiments and expenses limits the use of silico prediction, making 6mA prediction an ideal solution for tracking modifications in the genome. For the identification of 6mA, diversified techniques can be found in the literature. Initially, ultraviolet absorption spectra, paper chromatographic movement, and electrophoretic mobility were combined to represent a complete mechanism. Although this method was not efficacious enough to be used for detecting 6mA transformations in animals [9], this led to an introduction of another technique for identifying 6mA modification using a restriction enzyme, but this approach was only capable of identifying transformed adenines that are present in the target motifs [10].

For the detection of 6mA sites in prokaryotes and eukaryotes, numerous techniques were proposed such as single molecule real-time (SMRT) sequencing [11], methylated DNA immunoprecipitation sequencing [12], ultra-high performance liquid chromatography with mass spectrometry [1], and metabolically generated stable isotope-labeled deoxynucleoside code [13]. Chlamydomonas genes carry 84% N${}^{6}$-methyladenine modifications, which was identified after 6mA an immunoprecipitation sequencing experiment [14]. SMRT sequencing found out that adenines of methylated sites carry 2.8% of initial-diverged fungi [15]. Utilization of SMRT, 6mA immunoprecipitation, and mass spectrometry result in 0.2% of adenines being methylated [16].

The experimental techniques proved to be expensive and prolonged processes, therefore researchers tried to come up with computational techniques for prediction of DNA 6mA modifications. For this purpose, numerous prediction tools were proposed in the literature. iDNA6mA-PseKNC was the first ever N${}^{6}$-methyladenine modification prediction tool for the *Mus musculus* genome [17]. iDNA6mA-PseKNC proposed sequence sample formulation for feature extraction and employed six different classifiers to identify the modification. csDMA is another reported tool that predicts the modification in N${}^{6}$-adenine methylation, which used *K*-mer pattern, KSNPF frequency, nucleic shift density, binary code, and motif score matrix for extraction of the feature vector of the sequence [18]. Further, they deployed five different classifiers to evaluate the performance of the extracted feature set. Recently, 6mA-Finder was introduced as an online tool for predicting 6mA modification [19]. 6mA-Finder engaged seven sequence encoding schemes to get three types of physico-chemical features encoded. These encoded features were then embedded in seven different classifiers to evaluate the performance of encoded features. The i6mA-Pred is an identification tool for N${}^{6}$-methyladenine modification in the rice genome [20].

FastFeatGen is another tool present in the literature that predicts DNA N${}^{6}$ methyladenine sites [21]. FastFeatGen has used a parallel feature extraction technique followed by an exploratory feature selection algorithm to get the most relevant features. These features are then fed to Extra-Tree Classifier (ETC) for the prediction. Liang et al. proposed the i6mA-DNCP tool for the identification of 6mA sites [22]. i6mA-DNCP used optimized dinucleotide-based features with bagging classifier for the prediction model. Undoubtedly machine learning has illustrated high performance for many research problems, but the neural network has its benefits that need to be investigated for every research problem.

In recent years, Neural Network (NN)-based techniques, especially Convolution Neural Network (CNN), have shown tremendous improvement in many different research problems, e.g., in medical imaging [23,24] and bio-informatics [25,26,27], while the use of CNN for DNA-6mA modification identification is still in the infancy. Recently, a technique called iIM-CNN was reported by Wahab et al., which uses a CNN-based model for the N6-adenine methylation modification identification in genomes of different species [28]. The proposed CNN model in iIM-CNN carries two convolution layers with two max-pooling layers and a set of fully connected layers. iIM-CN showed high performance in prediction of N${}^{6}$-methyladenine modification, somehow still, a research space is available where many aspects of CNN can be explored more.

This article aims to provide a CNN and Long Short-Term Memory (LSTM)-based efficient tool named DNA6mA-MINT, for DNA 6mA modification identification. The proposed model uses CNN for feature extraction while LSTM gives optimal interpretation to those features. The proposed architecture demonstrates higher performance than the existing state-of-the-art techniques on the “combined-species”, *M. musculus* genome, and rice genome benchmark datasets. For better comparative analysis between DNA6mA-MINT and existing techniques, we have carried out performance analysis on 5- and 10-fold cross-validation. When compared with respective models available in the literature, Matthews Correlation Coefficient (MCC) for the “combined-species” benchmark dataset is noted with an increase of 20.83% for 5-fold cross-validation. The five steps are construction of dataset, encoding samples, constructing prediction model, evaluation of the proposed model, and establishing an online server. For the development of a useful and effective biological predictor, Chou’s 5-steps rule needs to be followed [29,30]. These steps were followed by the previous researchers as well [17,18,19,20,28]. This research article follows Chou’s 5-steps rule.

## 2. Benchmark Dataset

In this work, we used three datasets. The *M. musculus* genome database for DNA 6mA was proposed in 2018 by Feng et al. [17]. The dataset consists of 1934 samples for each positive and negative case. The 6mA sites available in the mouse genome were collected from MethSMRT database [31] with Gene Expression Omnibus (GEO) accession number GSE71866. Another dataset was on the rice genome, which was presented in 2019 by Chen et al. [20]. This dataset consists of 880 samples for each positive and negative case. The 6mA sites in rice genomes were provided by Zhou et al. [16] with GEO accession number GSE103145. Combining both aforementioned databases, a “combined-species” dataset is generated which contains 2768 samples for the positive cases and 2716 for negative cases. While the “combined-species” dataset did not contain sequence redundancy, which is eliminated by CD-HIT software [32], the rigorous sequence identity threshold was 0.80. Further, the dataset for training comprises 2214 positive samples and 2214 negative samples, while for the purpose of independent training 554 positive samples and 502 negative samples are taken into account. The length of all sequences in the datasets are 41 bp centered with the 6mA and non-6mA site.

## 3. Methodology

**Figure 1:**
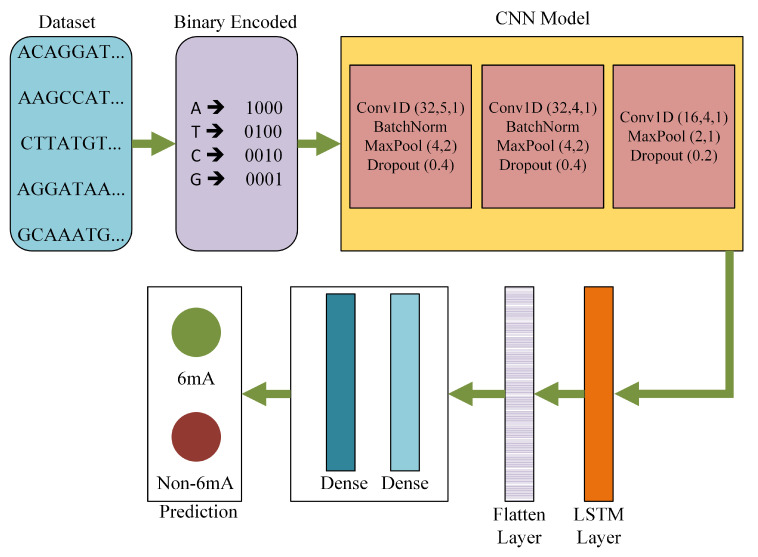
DNA6mA-MINT architecture for identification of DNA 6mA modification. Acronyms: Convolution 1 Dimension (Conv1D), BatchNormalization (BatchNorm), MaxPool (Max Pooling), Convolution Neural Network (CNN), Conv1d (number of filters, size of the filters, number of strides), MaxPool (pool size, number of strides), Dropout (ratio of features which needs to be discarded), and Long Short-Term Memory (LSTM).

The proposed architecture was an efficient deep learning-based model comprised of several convolution layers, hidden layers, LSTM layers, and dense layers. Figure 1 is a visual representation of DNA6mA-MINT. This model holds the capability of extracting critical features from the input raw sequence, which are then used to carry prediction. The input sequence carries a combination of 4 nucleotides, A, T, C, and G, as can be seen in the dataset block of Figure 1. The NNs work on the numerical data only, therefore an encoding scheme is required here which can effectively convert the sequence-based data to a numerical representation. For the said purpose, binary encoding was taken into account. Where A, T, C, and G are represented as (1, 0, 0, 0), (0, 1, 0, 0), (0, 0, 1, 0), (0, 0, 0, 1), respectively.

Table 1 shows the architecture details of DNA6mA-MINT. The DNA6mA-MINT includes three convolution layers that use different parameters to extract the features from the input binary encoded sequence. The first convolution layer uses 32 filters with a filter size of five, followed by another convolution layer which uses 32 different filters with a filter size of four. The last convolution layer uses 16 filters of size four. Features extracted by the first two convolution layers undergo Batch normalization, Max-pooling layer, and a dropout layer discarding 40% of features, while the features extracted by the last convolution undergo Max-pooling and dropout of 20%. The number of filters for the convolution layer with their filter size, Stride length, pool-size, and the dropout ratio is decided after hyperparameter tuning. Therefore, the selected values of the parameters were capable of giving the best performance from the model.

In CNN models a greater number of convolution layers represents the extraction of deeper features, but for the research problem under consideration, we cannot use more number of convolution layers, as by further increasing the convolution layers, the overfitting problem is observed. Using three convolution layers was an ideal solution to classify the input data we have, as this leads us to a high-performance architecture. All the convolution layers used ReLU as an activation function which eases the training process. At this stage, sigmoid or tanh are not used as an activation function, the reason being their vanishing gradient problem. The vanishing gradient problem makes the training process difficult, where ReLU solves this problem due to its unbounded nature.

The set of features extracted from the CNN model was fed into LSTM, which is a recurrent neural network (RNN). Here, the LSTM supports the sequence prediction. Therefore, the proposed model consists of two sub-models: the feature extractor which is the CNN model and the feature interpreter, which is the LSTM layer. In the proposed model, LSTM is used with a filter size of four, which is selected after hyperparameter tuning. The optimally interpreted feature set was converted to a single feature column by using a flattened layer. A single column feature set undergoes two dense layers with 32 and 1 neurons respectively to give the final classification output. The first dense layer uses the ReLU activation function while the second dense layer uses the sigmoid activation function. Sigmoid activation function makes the output range between 0 and 1 which is required for a binary classification problem. Below are the equations for ReLU and sigmoid functions.

(1) ReLU(z)=max(0,z)

(2) $$Sigmoid\left(z\right)=\frac{1}{1+exp(-z)}$$

DNA6mA-MINT is implemented on the Keras framework [33]. The output of the sigmoid activation function will be an input to the objective function. Binary cross-entropy is used as an objective function [34] and its equation is as follows,

(3) $$BCE=-y_{1}log\left(Sigmoid\left(z\right)\right)-\left(1-y_{1}\right)log\left(1-Sigmoid\left(z\right)\right)$$

where $y_{1}$ is the label for class sample. The loss can also be expressed as

(4) $$BCE=\left\{\begin{array}{cc} -log(Sigmoid(z)) & \mathrm{if}\;y_{1}=1\\ -log(1-Sigmoid(z)) & \mathrm{if}\;y_{1}=0 \end{array}\right.$$

Stochastic gradient descent is used for optimizing the objective function. The equation below is used for calculating stochastic gradient descent,

(5) $$\theta^{i+1}=\theta^{i}-\alpha \cdot {▽}_{\theta }Loss\left(\theta^{i},y\right)$$

where $\theta^{i}$ is the current estimation of θ at iteration ‘i’, α is the learning rate, and ${▽}_{\theta }Loss\left(\theta^{i},y\right)$ is computed gradient of the loss function.

Stochastic gradient descent reduces the computational complexity by achieving faster iterations [35]. In the optimization process, the learning rate and momentum were set to 0.004 and 0.9 respectively.

## 4. Figure of Merits

Evaluation of the DNA6mA-MINT is carried out using *k*-fold cross-validation where the value of *k* in our case is kept five and ten. In both cases, the whole dataset was divided into *k* subset. A single subset is chosen iteratively for the testing purpose where remaining subsets are used for training purposes. For the final performance estimation of the model, an average of *k*-trials is taken.

The figure of merits used in recent publications are listed with equations below,

(6) $$Sensitivity=TPR=\frac{TP}{TP+FN}$$

(7) $$Specificity=TNR=\frac{TN}{TN+FP}$$

(8) $$Accuracy=\frac{TP+TN}{TP+TN+FP+FN}$$

(9) $$MCC=\frac{TP\times TN-FP\times FN}{\sqrt{\left(TP+FP)(TP+FN)(TN+FP)(TN+FN\right)}}$$

where

TP = True Positive = 6mA correctly identified as 6mA

FP = False Positive = Non 6mA incorrectly identified as 6mA

TN = True Negative = Non 6mA correctly identified as Non 6mA

FN = False Negative = 6mA incorrectly identified as Non 6mA

Sensitivity, also known as True Positive Rate (TPR), is a statistical measure which calculates the ratio of positive samples identified as positive samples by the model. Specificity, also known as True Negative Rate (TNR), is also a statistical measure which calculates the ratio of negative samples identified as negative samples by the model. Accuracy measures the closeness of the model to the idle situation. While the Matthews correlation coefficient (MCC) depicts the quality of the model as a binary classifier, another figure of merit used in this study is the area under Receiver Operating Characteristics (auROC). It measures the performance of the model at various thresholds. The auROC indicates the capability of the model to distinguish two classes from each other.

## 5. Results and Discussion

The proposed model was evaluated on three datasets: *M. musculus* genome, rice genome, and “Combined-species”. The state-of-the-art techniques in the literature carried out their results either using 5-fold cross-validation or 10-fold cross-validation. Therefore, we validated DNA6mA-MINT by using both numbers of folds so that a better comparative analysis can be derived. Therefore, it is important to compare 5-fold cross-validation results with the models that have reported their results on 5-fold cross-validation. Similarly, 10-fold results should be compared with the 10-fold cross-validated model in the literature. A greater number of folds depicts higher performance, the reason being that by increasing the number of folds, the training dataset gets a higher ratio of the data which increases the model performance.

**Figure 2:**
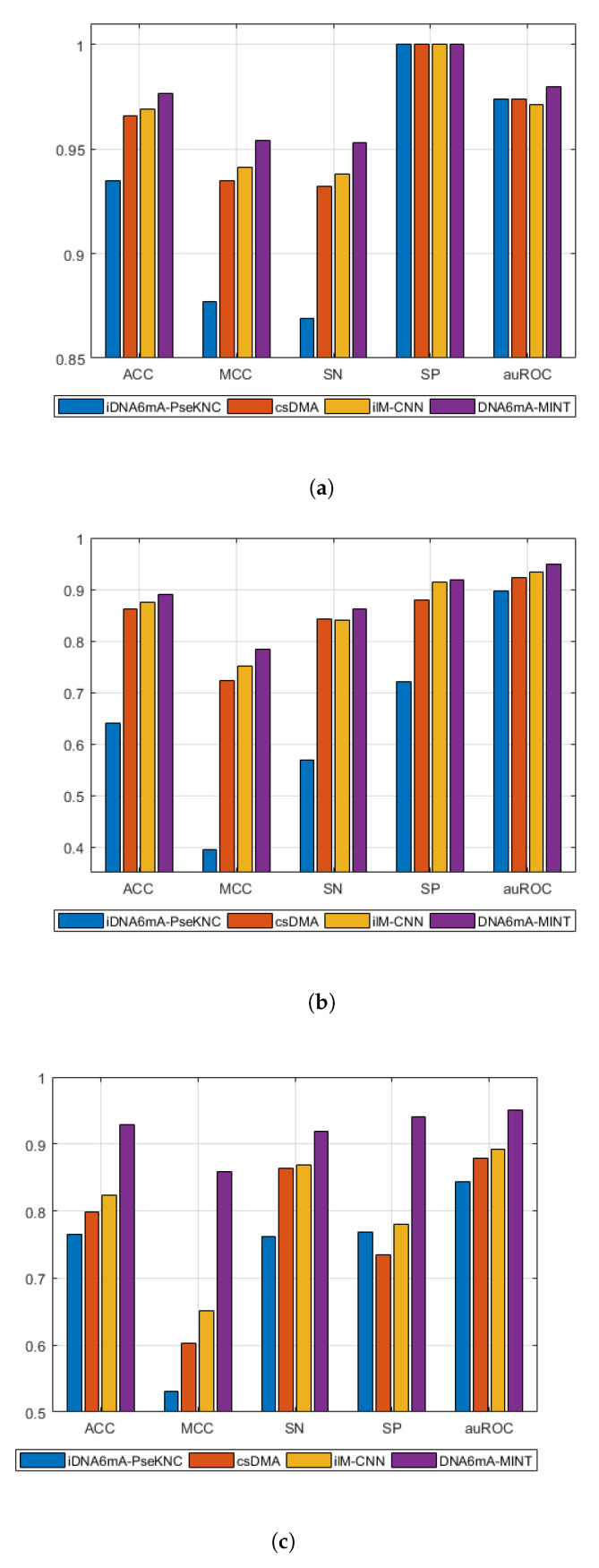
Graphical comparison of DNA6mA-MINT with state-of-the-art tools using five fold cross validation on different species. (**a**) *Mus musculus*, (**b**) Rice, (**c**) “Combined-species”. Acronyms are Sensitivity (SN), Specificity (SP), Accuracy (ACC), Matthews Correlation Coefficient (MCC), and area under the Receiver Operating Characteristics (auROC).

Table 2 shows a comparison of the proposed model with existing techniques, while Figure 2 shows the graphical visualization of performance differences between existing techniques and the proposed technique in this study. In the case of *M. musculus* genomes, the DNA6mA-MINT achieved high results in all figures of merit when compared with models validated on 5-fold cross-validation. On the other hand, compared on 10-fold cross-validation, the 6mA-Finder exhibits higher auROC then the proposed model. However, in all other figures of merit the proposed model remains higher in performance.

For Rice genomes with 5-fold cross-validation, the DNA6mA-MINT depicts an increase in all figures of merit, while in 10-fold cross-validation, 6mA-Finder has not reported results for all figures of merit, but the reported auROC achieved by 6mA-Finder is lower than that achieved by the proposed model in 10-fold cross-validation.

“Combined-species” is another benchmark dataset for the evaluation of the proposed model. In "combined-species", the proposed model has shown a tremendous increase in performance when compared with existing techniques. In 5-fold cross-validated models, the DNA6mA-MINT increased the sensitivity, specificity, accuracy, MCC, and AuROC by 4.92%, 16.09%, 10.55%, 20.83%, and 5.8%, respectively. For 10-fold cross-validation, the proposed model illustrated an increase of 3.93% in auROC when compared with 6mA-Finder. The sharp increase in MCC depicts the higher quality of the DNA6mA-MINT in comparison to existing state-of-the-art tools.

**Figure 3:**
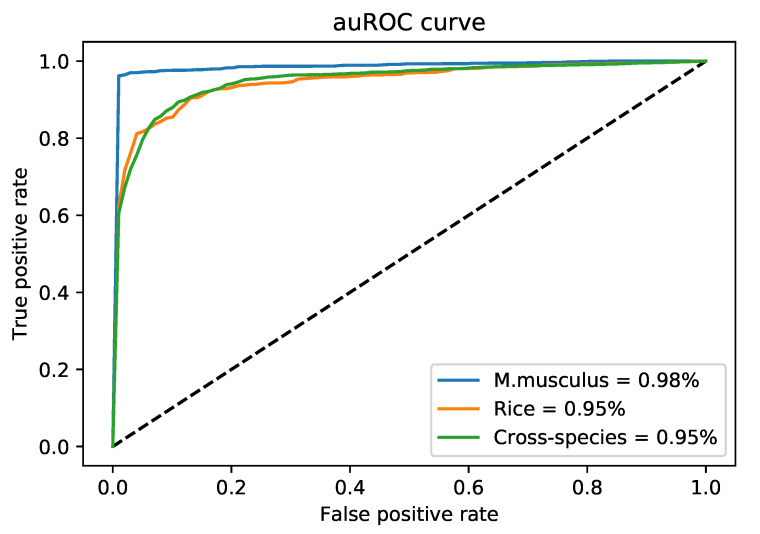
AuROC for *M. musculus*, Rice, and “Combined-species” genomes.

Figure 3 shows the auROC curves for three species. As can be determined by the curves, the proposed model curves are approaching the ideal scenario. Especially in the case of *M. musculus*, which is almost near to ideal. Upon evaluation of DNA6mA-MINT on the “combined-species” independent dataset with 10-fold cross-validation, a massive increase of 8.99% is observed in auROC. The 6mA Finder has reported 87.01% auROC while the proposed model has achieved 96% auROC for “combined-species” independent dataset. The high performance shown by the DNA6mA-MINT depicts the reliability of the proposed tool.

**Figure 4:**
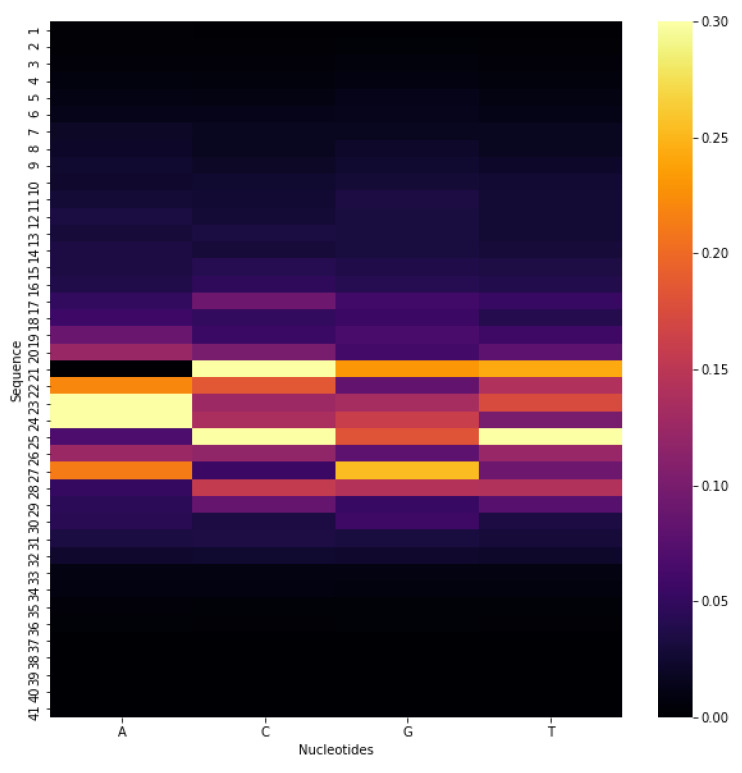
Heat Map to study the effect of mutation in model prediction.

For functional genomics, such an architecture should be used which can effectively model the DNA motifs with some insertion/deletion (indels). Keeping it in mind to unfold the quality of DNA6mA-MINT, the silico mutagenesis method is adopted. Nucleotides in the benchmark dataset are computationally mutated. The effect of this mutation in model prediction is studied. One by one the data at position “1-41” is mutated and the corresponding absolute difference is stored. Last, the averaged predicted score for all the mutations over all the sequences in the benchmark dataset is computed to construct the heat map. Figure 4 represents the constructed heat map illustrating the important position of the input sequence. As can be seen, the final prediction is more affected by the mutations occurring at the center of the sequence than the mutations happening on both sides of the sequence.

In order to study the generalization of DNA6mA-MINT we have prepared additional dataset for Rice genome (which is a part of our future work) from the NCBI Gene Expression Omnibus (https://www.ncbi.nlm.nih.gov/geo/) under the accession number GSE103145. We have prepared from this repository 10,000 positive sequences and 10,000 negative sequences that are not 6mA. Obtained values for sensitivity, specificity, and accuracy are 84.77, 82.78, and 83.76, respectively. The obtained results show that proposed model generalizes well to the new sequences.

## 6. Conclusions

DNA modification results in presiding form which is DNA N${}^{6}$-methyladenine (6mA). DNA-6mA identification is necessary to explore different biological functions. This study proposed an effective computational tool for the identification of DNA-6mA using a Neural Network framework. The proposed model uses a CNN for feature extraction followed by the LSTM layer, which gives interpretation of the high-dimensional feature vector so that they can be optimally utilized for classification of methylated or non-methylated sites. For comparison purpose results are computed on five and ten folds for three datasets. The proposed model outperformed the results achieved by existing state-of-the-art models in the case of all the datasets. The aim to introduce this model is to utilize it for different research fields working in the development of medicine and bioinformatics. For the said reason, a web server is created which is publicly available at: http://home.jbnu.ac.kr/NSCL/DNA6mA-MINT.htm.

## Figures and Tables

**Table 1:** Architecture details of DNA6mA-MINT.

Layer	Output Shape	Number of Parameters
Input	(41,4)	-
Conv1D (32,5,1)	(37,32)	672
Batch Normalization	(37,32)	128
Max Pooling (4,2)	(17,32)	0
Dropout (0.4)	(17,32)	0
Conv1D (32,4,1)	(14,32)	4128
Batch Normalization	(14,32)	128
Max Pooling (4,2)	(6,32)	0
Dropout (0.4)	(6,32)	0
Conv1D (16,4,1)	(3,16)	2064
Max Pooling (2,1)	(1,16)	0
Dropout (0.2)	(1,16)	0
LSTM	(1,4)	336
Flatten	4	0
Dense	32	160
Dense	1	33

**Table 2:** Performance comparison of DNA6mA-MINT with existing techniques on different species with 5- and 10-fold cross-validation.

Model	Species	Folds	SN	SP	ACC	MCC	auROC
iDNA6mA-PseKNC	*M. musculus*	5	0.869	1	0.935	0.877	0.974
	Rice	5	0.569	0.721	0.641	0.394	0.896
	Combined-species	5	0.762	0.769	0.765	0.531	0.844
csDMA	*M. musculus*	5	0.932	1	0.966	0.935	0.974
	Rice	5	0.842	0.880	0.861	0.723	0.923
	Combined-species	5	0.863	0.735	0.799	0.603	0.879
ilM-CNN	*M. musculus*	5	0.938	1	0.969	0.941	0.971
	Rice	5	0.841	0.914	0.875	0.752	0.934
	Combined-species	5	0.869	0.780	0.824	0.651	0.892
6mA-Finder	*M. musculus*	10	0.9349	1	0.9674	0.935	0.9954
	Rice	10	-	-	-	-	0.9394
	Combined-species	10	-	-	-	-	0.9207
DNA6mA-MINT	*M. musculus*	5	0.9531	1	0.9766	0.9543	0.980
	Rice	5	0.8621	0.9195	0.8908	0.7829	0.950
	Combined-species	5	0.9182	0.9409	0.9295	0.8593	0.950
DNA6mA-MINT	*M. musculus*	10	0.9427	1	0.9714	0.9444	0.98
	Rice	10	0.9425	0.908	0.9253	0.8511	0.950
	Combined-species	10	0.9318	0.9321	0.932	0.8639	0.960
